# Molecularly modified ultrathin Al_2_O_3_ layers as proton-conductive, oxygen-impermeable nanomembranes for catalytic surfaces

**DOI:** 10.1039/d5nr04262c

**Published:** 2026-02-05

**Authors:** Dalia Leon-Chaparro, Christos Englezos, Bastian Mei, Guido Mul, Georgios Katsoukis

**Affiliations:** a Department of Chemical Engineering, MESA+ Institute for Nanotechnology, Faculty of Science and Technology, University of Twente Drienerlolaan 5 7522 NB Enschede The Netherlands g.katsoukis@utwente.nl d.c.leonchaparro@utwente.nl c.englezos@utwente.nl g.mul@utwente.nl; b Laboratory of Industrial Chemistry, Ruhr-University Bochum Universitätsstraße 150 44801 Bochum Germany bastian.mei@rub.de

## Abstract

Ultrathin inorganic oxide coatings can improve selectivity in photo- and electrocatalysis, but they also bury active sites and impede transport of the desired reactants. Here we quantify proton and O_2_ permeability of 3–5 nm amorphous alumina (Al_2_O_3_) overlayers on poly-crystalline Pt using electrochemical impedance spectroscopy (EIS) and fourier-transform infrared reflection–absorption spectroscopy (FT-IRRAS). The apparent proton diffusivity amounts to ∼10^−13^ m^2^ s^−1^ in the atomic-layer-deposited (ALD) films. FT-IRRAS reveals hydrated AlOOH motifs whose presence correlates with the measured diffusion coefficients, highlighting their role as the dominant proton-transport pathways. The through-(Al_2_O_3_) film resistance is growing non-linear with thickness (17 → 37 Ω cm^2^ for 3 → 4 nm) and becomes close to infinity at 5 nm. Embedding oligo(ethylene glycol) chains within the alumina reduces the through-film resistance to 2.6 Ω cm^2^ at 3 nm. This is associated with enhancing proton access, albeit with a higher charge-transfer resistance (∼38 → 250 Ω cm^2^), consistent with diminished activity of the underlying Pt active sites. In O_2_-saturated electrolyte the total impedance increases and the diffusion contribution moves below the measurement threshold (1 Hz), indicating preserved oxygen-blocking character. Practically, this sets different design priorities. For high-current electrocatalysis, performance is governed by the overlayer's area-specific resistance, which can be improved by molecular functionalization. In low-current photocatalysis, the ohmic resistance penalty is small, so maintaining (or boosting) the intrinsic activity of buried active sites is more important to justify selectivity gains from O_2_ blocking.

## Abstract 2

Ultrathin inorganic oxide coatings can improve selectivity in photo- and electrocatalysis, but they also bury active sites and impede transport of the desired reactants.

## Graphical abstract

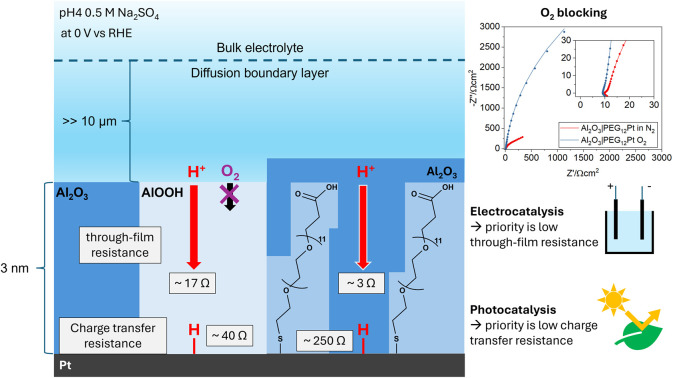

## Introduction

Electro- or photocatalytic H_2_ production from water is a promising sustainable alternative to steam methane reforming. However, these methodologies suffer from efficiency losses due to undesired reactions, catalyst surface restructuring, catalyst poisoning, and/or catalyst dissolution.^1–4^ In this context, ultrathin oxide electro- or photocatalyst overlayers have emerged as a promising concept^5^ to simultaneously enhance catalyst stability^6^ and improve selectivity by for example preventing the more favorable O_2_ reduction reaction in overall photocatalytic water splitting.^7–11^ These coatings can regulate the accessibility of reactants to the catalyst surface, poisoning and effectively suppress metal dissolution into the electrolyte.^11–13^ However, a critical trade-off arises in terms of activity. Such overlayers introduce an additional energy barrier, as they reduce reactant diffusivity toward the catalyst surface and the intrinsic activity of the buried active sites.^5,14,15^

To better understand the role of ultrathin oxide layers in electrocatalysis, it is important to gain insight into the mechanisms by which these layers influence performance. While previous studies have typically focused on overall activity trends, it remains not well understood to what extent such changes originate from diffusivity limitations *versus* altered reactivity of buried active sites.^5^ Esposito *et al.* reported on experimental methods and best practices for characterizing the transport and kinetic properties of species through oxide encapsulated electrocatalysts.^16^ Overlayer permeability can be determined in mass transfer limited current densities for example by using rotating disk electrode setups which has been successfully applied on ultrathin microporous silica membranes.^17^ Extracting quantitative parameters such as the through-film resistance of oxide overlayers, decoupled from diffusion boundary layer contributions, provides complementary information. By correlating these metrics with fabrication conditions, one can deepen the understanding of structure–property relationships and guide the rational design of overlayers for improved electrocatalytic performance.

Amorphous aluminum oxides are widely recognized as highly effective oxygen diffusion barriers, with diffusion coefficients in the order of 10^−20^–10^−22^ m^2^ s^−1^ for 100 nm films.^18^ This exceptionally low permeability renders them attractive for suppressing the oxygen reduction reaction when used to encapsulate catalysts. Ultrathin alumina layers are also used as passivation coatings, where they effectively mitigate interfacial defect states.^19–21^ They have also been employed to prevent dissolution of cathode materials in Li-ion batteries^22^ and to stabilize molecular dyes and catalysts on supports by hindering desorption, even at thicknesses as low as 1 nm.^23^ Recent work has further demonstrated that the performance of these ultrathin coatings can be tailored, either by embedding molecular relays to facilitate charge transport across insulating alumina layers.^24^ In our previous work on pulsed-laser-deposition of ultrathin alumina coatings on Pt electrodes,^14^ we demonstrated that the layer effectively suppresses O_2_ permeation, yet simultaneously buries proton-reduction active sites. At the same time, it exhibits a measurable proton diffusion coefficient of 10^−18^ m^2^ s^−1^. Interestingly, the layer becomes increasingly permeable to protons over time, suggesting dynamic structural or compositional changes that gradually improve proton accessibility.

Here, we employ electrochemical impedance spectroscopy to extract and isolate key transport and interfacial parameters – film resistance, pseudocapacitance, and the effective proton diffusion coefficient – of an ultrathin (3 to 5 nm) amorphous alumina overlayer deposited on Pt *via* atomic layer deposition (ALD). Using FT-IR reflection–absorption spectroscopy (FT-IRRAS), we establish a link between the structural features of the overlayer and its impedance. To further enhance proton transport, we grafted a self-assembled monolayer of an oligo(ethylene glycol) thiol with twelve repeating (PEG_12_) units onto Pt and subsequently deposited an alumina film *via* ALD. This strategy improves proton conductivity through alumina 7-fold, demonstrating the potential of incorporating functionalized self-assembled monolayers to tailor ion transport and boost conductivity in ultrathin oxide layers.

## Materials and methods

### Atomic layer deposition

The ALD alumina growth was conducted with a Veeco Savannah S100 Thermal ALD system (Veeco Instruments Inc. USA). The process temperature was maintained at 150 °C, with trimethylaluminum (elec. gr. 99.999+%-Al PURATREM, Strem Chemicals, Inc. USA) as the Al precursor and H_2_O (Milli-Q, 18.2 MΩ cm) as the oxidant. TMA and H_2_O were not pre-heated prior to delivery. Nitrogen as the carrier gas was flowed at 20 mL min^−1^. A complete cycle consisted of firstly a 0.015 s H_2_O dosing pulse followed by 5 s exposure, and secondly a 0.015 s TMA dosing pulse followed by 5 s exposure. Vacuum (100 mTorr with 20 mL min^−1^ N_2_ flow) was kept on continuously throughout the deposition. The estimated growth rate was ∼0.1 nm of Al_2_O_3_ per cycle at 150 °C.

### Magnetron sputtering

100 nm thin Pt electrodes were prepared on 2.5 × 2.5 cm^2^ Si substrates using a magnetron sputter coater (ATC Polaris, AJA International Inc., USA), equipped with a Pt target (99.99% purity, AJA International Inc., USA), powered by an RF power supply (Power source 0313GTC, T&C Power, USA). More details can be found in previous work.^25^

### Thiol-PEG_12_-acid self-assembled monolayers

Pt|Si wafers were solvent-cleaned (acetone, iso-propyl alcohol, water), dried, and used immediately. 1.0 mM Methyl terminated PEG_12_-thiol (ABCS, 95% purity) was dissolved in absolute ethanol and the solution was N_2_-purged for 10 min. Wafers were fully immersed and soaked overnight (12–18 h) at room temperature in the dark. Samples were rinsed and sonicated with ethanol, dried, and used immediately. Atomic layer deposition was performed directly on the PEG_12_-thiol coated Pt substrates.

### Electrochemical measurements

A BioLogic VMP3 Potentiostat was used. Electrochemical characterizations were carried out in a single compartment (15 mL) three electrode glass bottom cell using Al_2_O_3_ (3, 4, 5 nm)|Pt film (100 nm) on Si wafer support as working electrode (1 cm^2^ geometric area), a Pt wire counter electrode and an Ag/AgCl (3 M KCl) reference electrode. For electrochemical impedance spectroscopy (EIS) a sinusoidal AC wave with a ±10 mV amplitude was applied and measurements were done from 100 kHz to 0.1 Hz by collecting 10 points per decade. The acquired spectra were analysed using the ZView® software (Scribner Associates). Kramers–Kronig tests were done using Boukamp's approach.^26,27^

Prior to each experiment, the counter electrode was cleaned with 0.1 M sulfuric acid (ACS reagent, 95–98%, Sigma Aldrich) and Milli-Q water (18.2 MΩ cm). The electrolyte was prepared using Milli-Q water (18.2 MΩ cm) and sodium sulfate (ACS reagent, ≥99%, Sigma Aldrich). The pH of the solution was adjusted by adding sulfuric acid (ACS reagent, 95–98%, Sigma Aldrich). Before EIS measurements, the sample was immersed in the solution for 30 min under N_2_ saturation. All experiments were carried out at room temperature. The electrochemical active surface area (ECSA) of the samples was calculated from the integrated H_upd_ signal during CV cycling and using the reference factor of 210 µC cm^−2^ for polycrystalline Pt.^28^

### FT-IR reflection–absorption spectroscopy

A Bruker V80v was used, which is equipped with a liquid nitrogen cooled medium-band (12 000–600 cm^−1^) MCT detector and, an MIR polarizer (KRS-5) inserted into an automatic polarizer rotational unit. A variable angle reflection accessory (Bruker A513/Q) was used and set to 75 degrees. The resolution was set to 2 cm^−1^, the aperture was set to 1.5 mm. A set of 10 times 200 scans were collected for each p- and s-polarization. A plain 100 nm Pt layer sputtered on an atomically flat Si wafer served as a baseline. The final spectra were corrected for atmospheric water and baseline corrected using an endpoint straight line. More on quantification of spectral data can be found elsewhere.^29^

## Results and discussion

### Characterization of ALD deposited Al_2_O_3_ overlayers

**Fig. 1:**
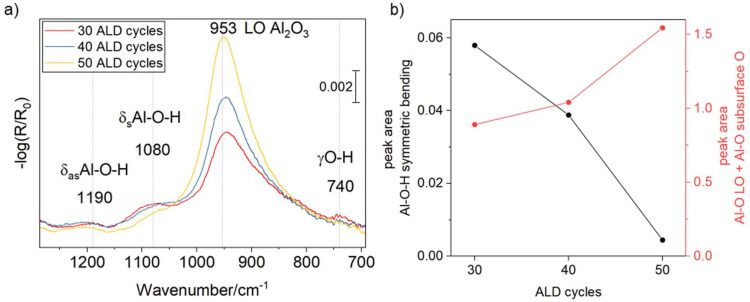
(a) FT-IRRA spectra of amorphous Al_2_O_3_ on Pt deposited *via* ALD. In addition to the dominant alumina longitudinal-optical (LO) mode, weak features attributable to hydrated alumina (Al–OH) are observed. (b) Evolution of the integrated area of the Al–O–H symmetric bending band with ALD cycle number, compared against the cumulative integrated area of the Al–O LO and Al–O (subsurface O) modes. The state of hydration decreases with increasing ALD cycles.

30 to 50 cycles of alumina were deposited on the Pt substrate at 150 °C *via* atomic layer deposition (ALD) using trimethylaluminium and water. In our work, we used FT-IRRAS to estimate the thickness of alumina layers, building on previous studies where the method was calibrated against scanning electron microscopy using thicker films.^14^ In addition, the thickness of the layers has been confirmed *via* X-ray reflectivity – see Fig. S1 and S2. Fig. 1a shows the corresponding FT-IRRA spectra for a 3, 4 and 5 nm ultrathin amorphous alumina layer on a polycrystalline Pt electrode. The LO (longitudinal optical) mode of alumina shifts from 947 to 953 cm^−1^ with increasing thickness of the layer which is typical for non-crystalline, amorphous ultrathin alumina films. We observe additional peaks that are most pronounced in the thinner coating at 1190 cm^−1^, 1080 cm^−1^ and 740 cm^−1^ which we assign to the asymmetric and symmetric Al–OH bending mode, and the Al–OH wagging mode of hydrated alumina, respectively, consistent with AlOOH formation.^30^ The peak deconvolution can be found in Fig. S3. Fig. 1b shows changes in the hydration state: the alumina-related peak area (red line) increases with the number of ALD cycles, whereas the intensity of the Al–OH wagging mode (black line) decreases with increasing ALD cycles.

### EIS of Al_2_O_3_ films (3, 4, 5 nm) on Pt

**Scheme 1:**
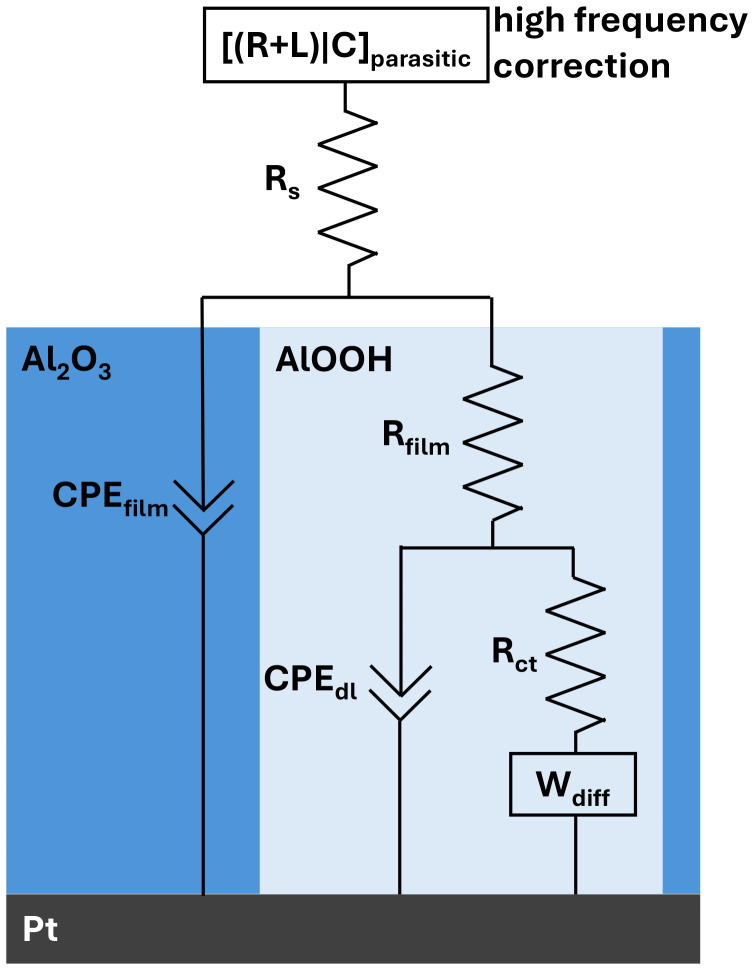
Equivalent circuit used to fit the EIS of Pt electrodes coated with an inert, nanoporous alumina overlayer. A small series inductance (to account for high-frequency wiring/lead effects) was included during fitting and corrected in the reported spectra. *R*_s_ is the solution resistance. *R*_film_ is the through-alumina-film protonic resistance and CPE_film_ captures the non-ideal capacitive charging of the hydrated, AlOOH-rich nanoporous network (pores shown in light blue). The buried catalyst/electrolyte interface inside the pores is modeled by a Randles branch with a semi-infinite Warburg element *W*_diff_, which accounts for charge-transfer kinetics and diffusion/mass transport within the pores.^31^

**Fig. 2:**
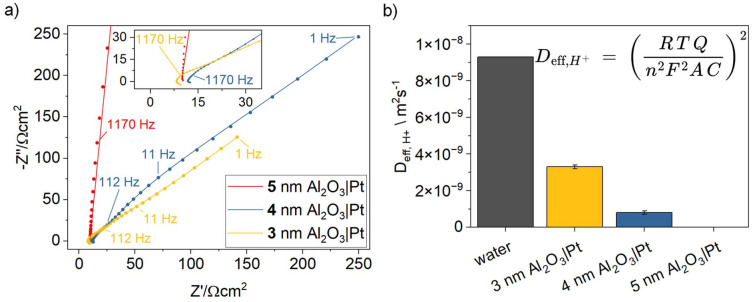
(a) EIS Nyquist plots (100 kHz to 1 Hz) for 3–5 nm ALD-grown Al_2_O_3_ on Pt, measured at 0 V *vs.* RHE in N_2_-purged 0.5 M Na_2_SO_4_ (pH 4). The inset shows a zoom into the high-frequency regime. (b) Effective proton diffusion coefficients extracted from the CPE_w_ parameter (*Q* in the formula shown) of the semi-infinite Warburg element. Calculations use the ideal gas constant *R*, temperature *T* = 298 K, number of electrons transferred *n* = 1, Faraday constant, electrode area *A* = 1 cm^2^, and *C* = 0.1 mM (concentration of protons).

To investigate the proton permeability of ultrathin Al_2_O_3_ films deposited on Pt *via* ALD, we performed electrochemical impedance spectroscopy (EIS) at 0 V *vs.* RHE (pH 4) in a N_2_-saturated electrolyte. Kramers–Kronig tests (Fig. S4) show that our data is valid from 100 kHz to 1 Hz. The equivalent circuit we used to fit the data is shown in Scheme 1. The corresponding CVs in Fig. S4 exhibit the typical response of dense ultrathin alumina-coated Pt electrodes, with no discernible H_upd_ features and complete suppression of the Fe^2+^/Fe^3+^ redox couple. The frequency range from 20 kHz to around 10 Hz contains two barely visible distorted semi-circles with a maximum inflection point at around 1000–500 Hz and 100 to 10 Hz. From 10 to 1 Hz, the plot follows a 45 degree transmission line (Fig. 2 incl. inset). For further quantitative analysis an “electrode coated with an inert porous layer” model has been used to fit the data.^31^

At the Pt–alumina interface localized at the end of a pore (*i.e.*, a hydrated alumina channel that enables proton transport, as discussed below), the corresponding impedance is given by a Randles circuit which is a parallel combination of the charge transfer resistance (*R*_ct_) in series with the semi-infinite Warburg element (*W*_diff_) and the pseudocapacitance of the double layer (CPE_dl_). Within the pore length, the electrolyte resistance is *R*_film_, and the insulating part of the coating is a pseudocapacitor (CPE_film_) which is in parallel with the impedance in the pore. *R*_film_ in practical terms stands for the additional ohmic potential required to transfer the proton through the film to the electrocatalyst surface and hence is a very powerful descriptor for the performance of an electrocatalyst coating. The bulk electrolyte resistance *R*_s_ is added in series with the previous impedance. For the high-frequency regime from 100 kHz to 20 kHz we implemented [inductance–series-resistance|parallel-capacitance] branch ([*L* − *R*|*C*]_parasitic_) to account for stray currents from wiring and contacting of the electrodes.^32^

Fig. 2a shows the EIS data for 3 nm (30 cycles), 4 nm (40 cycles), and 5 nm (50 cycles) ALD-grown Al_2_O_3_ layers on Pt and the determined fitting parameters are summarized in Table 1. Overall, the Nyquist plot shows a relatively large real and complex impedance. This is expected and arises from the combination of the relatively high pH of 4 (we are measuring the reductive proton adsorption at 0 V *vs.* RHE), the reduced number of accessible surface sites due to the alumina coating, and the intrinsically slower proton transport through amorphous alumina compared to the plain electrolyte. *R*_film_ doubles from 17.3 Ω to 35.7 Ω, when increasing the alumina thickness from 3 to 4 nm, which deviates from purely geometric scaling (where *R* would be linear proportional to thickness). The fact that we can resolve *R*_film_ arises from the corresponding RC time constant lying within the accessible high-frequency range of our EIS measurement (see Fig. 2a inset) slow enough to remain distinguishable and not merged into the inductive loop. CPE_film_ roughly doubles as well from 52 to 90 μΩ^−1^ cm^−2^ s^−*n*^ (*n*_film_ decreases from 0.94 to 0.87). We estimated the film's effective capacitance using the relation of Hirschorn *et al.*,^33,34^ obtaining 30.1 and 30.7 μF cm^−2^ for 3 and 4 nm, respectively. Importantly, a modest decrease in *n*_film_ has a large impact on the effective capacitance, because the deviation from ideal capacitive behavior strongly amplifies its influence in the Hirschorn relation. As corroborated by FT-IRRAS (Fig. 2b), we observe a progressive loss of film hydration with increasing film thickness. This is notable because both dehydration and increasing thickness should reduce the specific capacitance of the film.^35^ We therefore infer that the change in hydration level is minimal, yet it has a pronounced impact on proton permeability. This strong variation in *R*_film_ reflects the extreme sensitivity of proton transport to local hydration and defect density. At 5 nm, the Pt surface is fully passivated, and the EIS data are best described by a simple *R*∥CPE model with *R* → ∞ (red line Fig. 2a and Table 1, right column).

**Table 1:** Parameters obtained from fitting the EIS data from 100 kHz to 1 Hz with the equivalent circuit from Scheme 1. Transport parameters are also extracted. The effective proton diffusion coefficient in the electrolyte *D*_eff, H^+^_ (*R* is ideal gas constant, *T* is 298 K, *F* is the faraday constant, *n* is number of electrons transferred, *A* is electrode area, and H^+^ is proton concentration) is obtained from the semi-infinite Warburg coefficient. The effective diffusion coefficient of the alumina overlayer *D*_Al_2_O_3_, H^+^_ is derived from the fitted film resistance and film capacitance (*L* is the thickness of the alumina layer). For reference, the corresponding film conductivity *σ*_film_ is also reported

	3 nm Al_2_O_3_|Pt	4 nm Al_2_O_3_|Pt	5 nm Al_2_O_3_|Pt
*L*_parasitic_ [μH]	4.6	1.3	—
*R*_parasitic_ [Ω]	3.3	0.5	—
*C*_parasitic_ [μF cm^−2^]	0.1	0.9	—
*R*_s_ [Ω]	4.8	11.1	9.7
*R*_film_ [Ω]	17.3 ± 0.6	35.7 ± 1.6	>600 000
CPE_film_ [μΩ^−1^ cm^−2^ s^−*n*^]	52 ± 3	90 ± 3	2
*n*_film_	0.94	0.87	0.95
*R*_ct_ [Ω]	38 ± 4	150 ± 15	—
CPE_dl_ [μΩ^−1^ cm^−2^ s^−*n*^]	222 ± 37	185 ± 23	—
*n*_dl_	0.86	0.83	—
CPE_w_ [mΩ^−1^ cm^−2^ s^−0.5^]	2.16 ± 0.02	1.06 ± 0.08	—
*D*_eff, H^+^_ [10^−9^ m^2^ s^−1^]	3.3 ± 0.1	0.8 ± 0.1	—
*D*_Al_2_O_3_, H^+^_ [10^−13^ m^2^ s^−1^]	1.1	0.9	—
*σ*_film_ = *L*/*R*_film_*A* [nS cm^−1^]	0.1	0.1	—

The charge transfer resistance *R*_ct_ increases from ∼40 Ω at 3 nm to ∼150 Ω at 4 nm, corresponding to a decrease in exchange current density (*R*_ct_ is proportional to *i*_0_^−1^).^36^ A charge-transfer resistance in the tens of ohms is consistent with expectations for Pt at pH 4. On an ideal Pt surface, *R*_ct_ is only a few milliohms at pH 0,^37^ where the exchange current density is very high. Because i_0_ scales linearly with proton concentration, it decreases by four orders of magnitude at pH 4, leading to a corresponding four-order increase in *R*_ct_. Thus, an *R*_ct_ of ∼50 mΩ at pH 0 translates to ∼50 Ω at pH 4.^38^ As a consequence, the ALD alumina layer is barely affecting the activity of the Pt electrode. The additional rise in *R*_ct_ from 3 to 4 nm alumina, likely reflects either a reduction in electrochemically active surface area or buried-interface effects that shift H adsorption thermodynamics away from the optimum for H_upd_. The effective double-layer capacitance, derived from the pseudocapacitive CPE_dl_ decreases from 69 to 49 μF cm^−2^, consistent with the presence of insulating oxides on the electrode surfaces.

The low-frequency tail of the EIS spectra is well described by a semi-infinite Warburg element, from which the Warburg coefficient (CPE_w_) can be extracted. This enables determination of the effective proton diffusion coefficient within the whole diffusion double layer, incorporating contributions from both the electrolyte and the ultrathin alumina film. For comparison, the diffusion coefficient of bulk water is also included. Notably, 3 and 4 nm alumina layers reduce the apparent effective proton diffusivity by approximately factors of 3 and 10 respectively, underscoring the pronounced impact of ultrathin alumina films on proton transport. To disentangle the alumina contribution from the apparent diffusion coefficient, we use the film resistance *R*_film_ (see Table 2). The proton diffusion coefficient through the alumina is then calculated as (eqn (1)):1
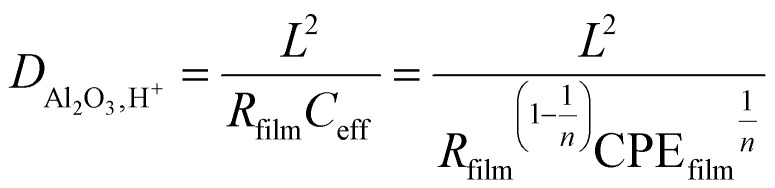
where *L* is the thickness of the alumina layer and *n* is *n*_film_.^39^ Using this approach, we obtain a proton diffusion coefficient in the order of 10^−13^ m^2^ s^−1^ for both the 3 and 4 nm layer. This value is significantly higher (5 orders of magnitude) than what we previously determined for pulsed-laser-deposited amorphous alumina ultrathin films on Pt (10^−18^ m^2^ s^−1^).^14^ Secondary ion mass spectrometry studies on hydrated alumina ultrathin films have reached the same conclusion, namely that hydration enables markedly faster proton transport.^40^ The reduction in overall proton transport arises from a decreased number of transport pathways in ALD deposited Al_2_O_3_, suggesting that hydrated alumina channels constitute the predominant pathway for proton diffusion toward the electrode surface. In other words, the proton diffusion coefficient is identical for the 3 and 4 nm films because the intrinsic diffusion mechanism along Al–OH pathways remains unchanged, while the increase in *R*_film_ reflects a reduction in the density of these pathways rather than a change in their mobility.

**Table 2:** Parameters obtained from fitting the time-resolved EIS data from 100 kHz to 1 Hz with the equivalent circuit from Scheme 1

Al_2_O_3_|PEG_12_Pt	4 min	8 min	12 min	16 min	20 min	24 min	28 min
*L*_parasitic_ [μH]	4.7	2.5	2.0	1.7	1.6	1.6	1.8
*R*_parasitic_ [Ω]	4.9	0.9	0.5	0.4	0.3	0.3	0.4
*C*_parasitic_ [μF cm^−2^]	0.1	0.4	0.6	0.7	0.8	0.8	0.7
*R*_s_ [Ω]	3.3	7.8	8.5	8.9	9.2	9.3	9.3
*R*_film_ [Ω]	12 ± 0.5	6.1 ± 0.3	4.3 ± 0.3	3.4 ± 0.2	2.8 ± 0.2	2.6 ± 0.3	2.6 ± 0.3
CPE_film_ [μΩ^−1^ cm^−2^ s^−*n*^]	40 ± 9	32 ± 9	27 ± 10	24 ± 11	25 ± 12	27 ± 14	31 ± 17
*n*_film_	0.75	0.79	0.83	0.87	0.90	0.91	0.92
*R*_ct_ [Ω]	703 ± 12	558 ± 8	467 ± 7	398 ± 5	341 ± 4	295 ± 3	255 ± 2
CPE_dl_ [μΩ^−1^ cm^−2^ s^−*n*^]	110 ± 5	100 ± 5	98 ± 6	97 ± 8	95 ± 10	93 ± 13	89 ± 17
*n*_dl_	0.94	0.93	0.93	0.92	0.92	0.91	0.91
CPE_w_ [mΩ^−1^ cm^−2^ s^−0.5^]	1.5	1.31	1.22	1.16	1.11	1.07	1.02
*D*_eff, H^+^_ [10^−9^ m^2^ s^−1^]	1.6	1.2	1.1	1.0	0.9	0.8	0.7
*D*_Al_2_O_3_, H^+^_ [10^−13^ m^2^ s^−1^]	0.4	0.5	0.7	1.1	1.4	1.4	1.4
*σ*_film_ [10^−9^ S cm^−1^]	0.2	0.5	0.7	0.9	1.1	1.2	1.2

Further, the alumina film behaves as an ultrathin membrane that introduces an additional ohmic resistance, *R*_film_. In PEM water electrolysis (pH 0), the HER overpotential on a Pt cathode is practically negligible even at 2 A cm^−2^ (<5 mV).^41^ Likewise, the effective proton-transport sheet resistance of a Pt/C electrode can be as low as 0.0025 Ω cm^2^, leading to insignificant losses. By contrast, a 3 nm alumina overlayer adds 17.3 Ω cm^2^ of proton-transport resistance which is approximately 7000-fold higher. These figures underscore the sizable energetic penalty imposed by the oxide layer despite its nanoscale thickness and membrane-like role. Nevertheless, because the transport properties of ultrathin oxides can be engineered (*e.g.*, *via* AlOOH or molecular doping), meaningful gains remain feasible. Motivated by this, we embedded vertically aligned oligo(ethylene glycol) molecules into the alumina matrix to study their impact on the film resistance. This approach is inspired by nature, which has ion conducting channels embedded into its membranes.

### EIS of Al_2_O_3_ films on PEG_12_-Pt

**Fig. 3:**
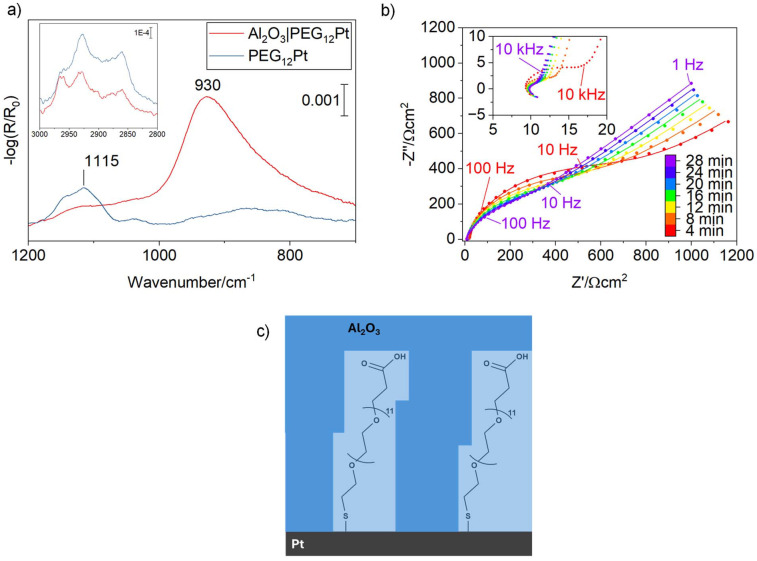
(a) FT-IRRA spectra of a PEG_10_-thiol self-assembled monolayer on Pt before and after deposition of an effective 1.4 nm amorphous Al_2_O_3_ overlayer by ALD. The band at 1115 cm^−1^ corresponds to the C–O–C stretching vibration of the PEG_12_ moiety, which is slightly reduced in intensity after ALD. The inset shows the C–H stretching region, confirming that the PEG_12_ layer remains present at the surface, although morphological changes lead to a modified intensity ratio of the bands. (b) Temporal evolution of the EIS responses of the PEG_12_-embedded alumina coated Pt electrode. Inset shows a zoom into the high-frequency regime. (c) Schematic of PEG_12_ thiol grafted on Pt and embedded inside alumina.

Oligo(ethylene glycol) thiol molecules with twelve repeating units (PEG_12_ thiol) were grafted onto Pt electrodes by immersing the electrodes overnight in a 1 mM ethanol solution of PEG_12_ thiol. In their extended conformation, these oligomers span approximately 3 nm. Upon atomic layer deposition of 30 cycles of trimethylaluminum (TMA), we observe in Fig. 3a that the intensity of the Al–O longitudinal optical (LO) mode is only 54% of that measured for alumina deposited directly on bare Pt (Fig. 1a). Because the vibrational intensity scales linearly with the concentration of Al–O species, this indicates a reduced effective alumina thickness on the PEG12-thiol-modified surface, corresponding to approximately 1.4 nm rather than 3 nm.

Choi *et al.* investigated the molecular mechanism of alumina ALD on self-assembled monolayers using sum-frequency generation (SFG) spectroscopy and showed that *gauche* defects within the SAM enable precursor physisorption, followed by condensation and growth of alumina.^42^ The oxide subsequently propagates toward the substrate, encapsulating the SAM layer.

We also observe that the C–O–C stretching vibration doublet at 1115 cm^−1^ exhibits noticeably reduced intensity following alumina deposition. This attenuation may result either from partial decomposition due to the chemical reactivity of the alumina precursor at 150 °C or from a change in the molecular tilt angle, which alters the orientation of the transition dipole moment relative to the surface normal. Analysis of the C–H stretching region shows that the characteristic CH_2_ stretching modes remain present, with only slight changes in their relative intensities. This suggests that the PEG_12_ backbone remains largely intact and that the observed changes likely stem from deviations in orientation rather than molecular degradation.

Fig. 3b shows the time-resolved EIS data obtained at 0 V *vs.* RHE for the oligomer-embedded 3 nm alumina modified Pt electrode (Fig. S5 shows the cyclic voltammogram). In comparison to the unmodified alumina-coated electrodes, the spectra undergo noticeable changes, indicating an observable activation process (Fig. S6 shows the KK-tests demonstrating that the out-of-equilibrium changes are below 1 Hz, enabling proper fitting of the data). We attribute these changes to cleavage of the Pt–S bond (Fig. S7), leading to progressive removal of the anchoring groups as a result of the measurement conditions as the applied potential (0 V *vs.* RHE) is negative of the stripping potential of thiols on Pt (see eqn (2)):^43^

2 Pt–SR + e^−^ ⇒ Pt + S^−^–R

Consistent with the unblocking of catalytic Pt sites, the charge-transfer resistance *R*_ct_ decreases from 700 to 250 Ω over the course of the impedance measurement (Table 3). While partial desorption under applied potential cannot be fully excluded, a gradual evolution of *R*_film_ over this timescale is inconsistent with a purely desorption-driven process. Nevertheless, *R*_ct_ remains substantially larger than for the unmodified alumina-coated electrodes, indicating that the buried interface exhibits limited intrinsic activity and/or incomplete site accessibility. In parallel, the film resistance decreases from 12.2 to 2.6 Ω, which is sevenfold lower than for unmodified alumina overlayers.

**Table 3:** EIS fitting parameter using the model in Fig. 4b

	3 nm Al_2_O_3_|Pt in O_2_
*L*_parasitic_ [μH]	2.7
*R*_parasitic_ [Ω]	3.3
*C*_parasitic_[μF cm^−2^]	0.8
*R*_s_ [Ω]	5.2
*R*_ct_ [Ω]	105 ± 74
CPE_dl_ [μΩ^−1^ cm^−2^ s^−*n*^]	73 ± 3
*n*_dl_	0.92
*R*_*W*_0__ [Ω]	1077 ± 125
*τ*_*W*_0__ [s^−1^]	0.04
*Φ*_*W*_0__	0.39

Because the oligomers are encapsulated within the alumina matrix, they unlikely dissolve into the electrolyte once stripped. Their removal would require substantial structural defects or pinholes in the alumina layer, conditions that would manifest in a breakdown of the film's barrier properties and result in an EIS response resembling that of bare Pt in aqueous electrolyte, which is not observed. *In situ* electrochemical IR reflection–absorption spectroscopic studies are underway to shed more light into the structural reconfigurations of self-assembled monolayers encapsulated within oxide matrices.

## Discussion

In a previous study, the proton permeability of PLD amorphous alumina layers prepared by pulsed laser deposition (PLD) on Pt was investigated.^14^ A key observation was that the impedance spectra could only be adequately fitted with a Randle's circuit containing a finite-length Warburg element with reflective boundary conditions. In contrast, the ALD samples examined in this study are not characterised by this model, but are most accurately reproduced by the equivalent circuit depicted in Scheme 1, which incorporates a semi-infinite Warburg contribution. Here, transport through the ultrathin alumina layer is described by the film resistance *R*_film_ while the Warburg element reflects proton diffusion within the whole diffuse boundary layer rather than across the oxide layer. This finding suggests that the effective transport for protons accessing Pt sites differs between PLD and ALD alumina overlayers.

The distinction can be rationalized by considering the alternating current diffusion penetration depth, 
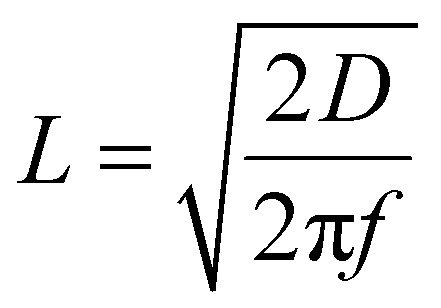
 which sets the distance over which concentration oscillations extend at a given frequency. For PLD alumina, the effective proton diffusivity is very low (*D* ∼ 10^−18^ m^2^ s^−1^). At the lowest measured frequency (1 Hz), *L* is 0.5 nm which is much smaller than the film thickness of 3–5 nm. Consequently, the alternating current (AC) perturbation only probes a shallow region of the oxide, and protons cannot explore the full thickness within the experimental timescale. In terms of electromechanical impedance, this is consistent with diffusion-limited oscillation phenomena, characterized by a reflective boundary condition, due to the confinement of the oscillating concentration field within the oxide layer, without reaching an absorbing sink at the buried Pt. In other words, A finite-length diffusion response is only expected when proton transport through the alumina layer is slow enough to limit the system's response to the AC perturbation, which does not apply to the 3 or 4 nm thin hydrated ALD-grown films.

For ALD alumina, the effective diffusivity is 5 orders of magnitude higher (*D* ∼ 10^−13^ m^2^ s^−1^). Under the same conditions, the penetration depth is hundreds of nanometers. In this regime the oxide layer is effectively transparent on the timescale of the AC perturbation. The impedance response therefore maintains the characteristic 45° Warburg slope down to low frequencies. We attribute this pronounced difference to the higher degree of hydration in the alumina films, as discussed above. It should be noted, however, that the properties of ultrathin alumina layers are highly sensitive to the deposition parameters in both PLD and ALD, offering considerable scope for tunability. Alumina also appears widely in electrocatalysis as a porous support material, where its surface chemistry itself can influence catalytic performance.^44^

As noted above, the apparent activity of Pt is reduced by about one order of magnitude (*e.g.*, *R*_ct_ ∼ 250 Ω *vs.* ∼50 Ω for ideal Pt at pH 4). In addition, the PEG_12_ modified ALD alumina introduces an area-specific through-film resistance of *R*_film_*ca.* 2.6 Ω cm^2^. Under the same Tafel kinetics (30–120 mV dec^−1^ on polycrystalline Pt), a 10 times drop in activity requires “one Tafel slope” of extra overpotential (*i.e.*, 30–120 mV) to reach the same current density. At practical currents the ohmic penalty of the film dominates: the IR-drop is Δ*V*_ohmic_ = *jR*_film_, so at *j* = 1 A cm^−2^ the overlayer alone increases the ohmic potential by ∼2.6 V. This underscores that minimizing *R*_film_ is far more critical than modest gains in interfacial kinetics for coated-electrode performance. Nevertheless, in photocatalytic architectures, ultrathin alumina (and related oxide) overlayers can play a disproportionately beneficial role since current densities are usually at around 10–20 mA cm^−2^, resulting into film resistance overpotential losses of only 26 mV for this example.^45^

**Fig. 4:**
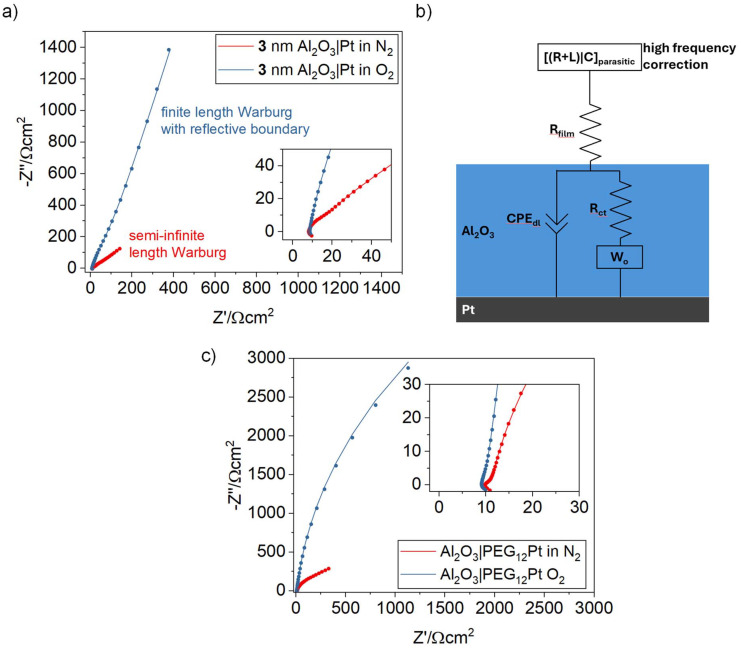
(a) EIS Nyquist plots (100 kHz to 1 Hz) for 3 nm ALD-grown Al_2_O_3_ on Pt, measured at 0 V *vs.* RHE in N_2_-purged and O_2_ 0.5 M Na_2_SO_4_ (pH 4). (b) Equivalent circuit model used to fit the EIS data in O_2_ saturated electrolyte. *W*_0_ is the generalized finite-length Warburg element with reflective boundary. (c) EIS Nyquist plots (100 kHz to 1 Hz) for 3 nm PEG_12_ modified Al_2_O_3_ on Pt, measured at 0 V *vs.* RHE in N_2_-purged and O_2_ 0.5 M Na_2_SO_4_ (pH 4). Note, that the equivalent circuit model used to fit the PEG_10_ modified Al_2_O_3_ on Pt in O_2_ is the one in Scheme 1. However, the time constant converged always towards infinity, showing that the diffusion part is not within the time-window of the measurement.

An important aspect is to compare proton permeability of the alumina layers to O_2_ permeability. Fig. 4 shows the EIS Nyquist plots of the 3 nm alumina-coated Pt electrode measured at 0 V *vs.* RHE (pH 4) under N_2_ (from Fig. 2) and O_2_ saturation. We initially anticipated being able to quantify O_2_ diffusivity, since 0 V *vs.* RHE corresponds to a 1.23 V overpotential for oxygen reduction, which should provide a much stronger driving force than that for proton underpotential deposition and, in principle, open a parallel faradaic channel in the EIS response that would lower the impedance. However, the impedance under O_2_ exceeds that observed under N_2_, indicating that O_2_ not only fails to permeate the alumina layer even at a 1.23 V overpotential but also further enhances its apparent barrier character (see Table 4 for the parameters obtained by fitting a Randle's circuit with a finite-length Warburg element with reflective boundary, Fig. 4b). The PEG_12_ modified alumina coatings shows similar behavior where the total impedance at 0 *vs.* RHE in O_2_ exceeds that of the N_2_ purged sample. The time constant of the semi-infinte Warburg element converged to infinity, pointing to the fact that we do not capture any diffusion process when measuring down to 1 Hz. At present, the origin of the higher total impedance in O_2_-saturated electrolyte remains unresolved and warrants further study into how dissolved O_2_ perturbs local alumina hydration and proton transport. From cyclic voltammetry in O_2_ saturated atmosphere (Fig. S8) some degree of O_2_ reduction can be observed, which may lead towards consuming protons *via* the O_2_ reduction pathway instead of the less resistive H_upd_, changing the EIS response. Crucially, both unmodified and PEG_12_-modified alumina retain effective O_2_ blocking character compared to uncoated Pt at 0 V *vs.* RHE.

**Table 4:** EIS fitting parameter using the model in Fig. 4b

	3 nm Al_2_O_3_|PEG_10_Pt in O_2_
*L*_parasitic_ [μH]	2.3
*R*_parasitic_ [Ω]	1.1
*C*_parasitic_ [μF cm^−2^]	0.3
*R*_s_ [Ω]	7.9
*R*_film_ [Ω]	21 ± 0.5
CPE_film_ [μW^−1^ cm^−2^ s^−*n*^]	34 ± 4
*n*_film_	0.96
*R*_ct_ [Ω]	9740 ± 75
CPE_dl_ [μW^−1^ cm^−2^ s^−*n*^]	13 ± 4
*n*_dl_	0.93

## Conclusions

In summary, we quantified the proton and O_2_ permeability of ultrathin ALD-grown alumina overlayers on Pt electrodes, with and without embedded oligo(ethylene glycol) monolayers. Our results establish that hydrated AlOOH phases form the predominant proton transport channels, yielding diffusivities on the order of 10^−13^ m^2^ s^−1^ which is five orders of magnitude higher than in PLD-grown alumina. Interestingly, the charge transfer resistance of the 3 nm alumina coated in the tens of Ohm cm^2^ range shows that it is not the activity of buried active sites that reduce proton reduction. By applying an inert-porous-layer impedance model, we deconvoluted the through-film resistance, showing that nm-thin alumina introduces tens of ohms of additional proton-transport resistance, a non-negligible barrier for HER. Hence, for high-current electrocatalysis performance is governed by the overlayer's area-specific resistance whereas in low-current photocatalysis the ohmic resistance penalty is small, so maintaining (or boosting) the intrinsic activity of buried active sites is more important to justify selectivity gains.

Embedding oligo(ethylene glycol) into the oxide matrix reduces this resistance sevenfold, though at the expense of higher charge-transfer resistance, illustrating the central trade-off between improved transport and buried-site activity. Finally, EIS in O_2_-saturated electrolyte reveals that modified and unmodified alumina maintains complete oxygen-blocking functionality, manifested as an overall increase in total impedance. Under these conditions, both charge-transfer and through-film resistances rise, while the diffusion contribution shifts below the accessible frequency window (<1 Hz).

These findings demonstrate that the ionic transport properties of dense amorphous alumina can be tuned by hydration and molecular functionalization, providing design rules for ultrathin oxide catalyst overlayers. More broadly, embedding molecular ion-conducting motifs into inorganic membranes offers a strategy to reconcile stability, selectivity, and activity in coated electro- and photocatalysts, not only for hydrogen evolution but also for other small-molecule conversions.

## Author contributions

The manuscript was written through contributions of all authors. All authors have given approval to the final version of the manuscript.

## Conflicts of interest

There are no conflicts to declare.

## Supplementary Material

NR-018-D5NR04262C-s001

## Data Availability

The raw and processed data supporting this study will be publicly available on Zenodo upon acceptance of the manuscript, with a DOI assigned at that time. All data presented in the figures are derived from this dataset. Supplementary information (SI) is available. The supplementary information contains X-ray reflectivity data for determining the thickness of the alumina layers, peak deconvolution of FT-IRRAS data, Kramers-Kronigs test of the EIS data, and cyclic voltammogramms of the samples investigated. See DOI: https://doi.org/10.1039/d5nr04262c.

## References

[cit1] Jerkiewicz G. (2010). Electrochemical Hydrogen Adsorption and Absorption. Part 1: Under-Potential Deposition of Hydrogen. Electrocatalysis.

[cit2] Zhai W., Ma Y., Chen D., Ho J. C., Dai Z., Qu Y. (2022). Recent Progress on the Long–term Stability of Hydrogen Evolution Reaction Electrocatalysts. InfoMat.

[cit3] Zhang X., Xiao Z., Jiao L., Wu H., Tan Y., Lin J., Yuan D., Wang Y. (2024). Molecular Engineering of Methylated Sulfone–Based Covalent Organic Frameworks for Back–Reaction Inhibited Photocatalytic Overall Water Splitting. Angew. Chem..

[cit4] Bhardwaj A. A., Vos J. G., Beatty M. E. S., Baxter A. F., Koper M. T. M., Yip N. Y., Esposito D. V. (2021). Ultrathin Silicon Oxide Overlayers Enable Selective Oxygen Evolution from Acidic and Unbuffered PH-Neutral Seawater. ACS Catal..

[cit5] Zhou J., Ming F., Liang H. (2025). Application of functional coatings in water electrolyzers and fuel cells. Nanoscale.

[cit6] Smiljanić M., Panić S., Bele M., Ruiz-Zepeda F., Pavko L., Gašparič L., Kokalj A., Gaberšček M., Hodnik N. (2022). Improving the HER Activity and Stability of Pt Nanoparticles by Titanium Oxynitride Support. ACS Catal..

[cit7] Takata T., Pan C., Nakabayashi M., Shibata N., Domen K. (2015). Fabrication of a Core–Shell-Type Photocatalyst via Photodeposition of Group IV and V Transition Metal Oxyhydroxides: An Effective Surface Modification Method for Overall Water Splitting. J. Am. Chem. Soc..

[cit8] Qureshi M., Shinagawa T., Tsiapis N., Takanabe K. (2017). Exclusive Hydrogen Generation by Electrocatalysts Coated with an Amorphous Chromium-Based Layer Achieving Efficient Overall Water Splitting. ACS Sustainable Chem. Eng..

[cit9] Worsley M., Smulders V., Mei B. (2022). Controlled Synthesis of Chromium-Oxide-Based Protective Layers on Pt: Influence of Layer Thickness on Selectivity. Catalysts.

[cit10] Jo W. J., Katsoukis G., Frei H. (2020). Ultrathin Amorphous Silica Membrane Enhances Proton Transfer across Solid–to–Solid Interfaces of Stacked Metal Oxide Nanolayers While Blocking Oxygen. Adv. Funct. Mater..

[cit11] Han K., Haiber D. M., Knöppel J., Lievens C., Cherevko S., Crozier P., Mul G., Mei B. (2021). CrO_x_-Mediated Performance Enhancement of Ni/NiO-Mg:SrTiO_3_ in Photocatalytic Water Splitting. ACS Catal..

[cit12] Chaveanghong S., Nakamura T., Takagi Y., Cagnon B., Uruga T., Tada M., Iwasawa Y., Yokoyama T. (2021). Sulfur Poisoning of Pt and PtCo Anode and Cathode Catalysts in Polymer Electrolyte Fuel Cells Studied by Operando near Ambient Pressure Hard X-Ray Photoelectron Spectroscopy. Phys. Chem. Chem. Phys..

[cit13] Jackson C., Raymakers L. F. J. M., Mulder M. J. J., Kucernak A. R. J. (2020). Assessing Electrocatalyst Hydrogen Activity and CO Tolerance: Comparison of Performance Obtained Using the High Mass Transport ‘Floating Electrode’ Technique and in Electrochemical Hydrogen Pumps. Appl. Catal., B.

[cit14] Leon-Chaparro D., Nguyen M. D., Baeumer C., Mul G., Katsoukis G. (2025). Mechanistic Insights Into Proton and Oxygen Transport Through Ultrathin Amorphous Al_2_O_3_ and Al_2_O_3_–SiO_2_ Electrocatalyst Overlayers. Adv. Mater. Interfaces.

[cit15] Smulders V., Gomes A. S. O., Simic N., Mei B., Mul G. (2021). Mixed Chromate and Molybdate Additives for Cathodic Enhancement in the Chlorate Process. Electrocatalysis.

[cit16] Esposito D. V. , Guilimondi V., Vos J. G. and Koper M. T. M., in Ultrathin Oxide Layers for Solar and Electrocatalytic Systems, ed. H. Frei and D. Esposito, The Royal Society of Chemistry, 2022, ch. 7, pp. 167–209. 10.1039/9781839163708-00167

[cit17] Bau J. A., Takanabe K. (2017). Ultrathin Microporous SiO_2_ Membranes Photodeposited on Hydrogen Evolving Catalysts Enabling Overall Water Splitting. ACS Catal..

[cit18] Nakamura R., Toda T., Tsukui S., Tane M., Ishimaru M., Suzuki T., Nakajima H. (2014). Diffusion of Oxygen in Amorphous Al_2_O_3_, Ta_2_O_5_, and Nb_2_O_5_. J. Appl. Phys..

[cit19] Banerjee S., Das M. K. (2021). A Review of Al_2_O_3_ as Surface Passivation Material with Relevant Process Technologies on C-Si Solar Cell. Opt. Quantum Electron..

[cit20] Eberhart M. S., Wang D., Sampaio R. N., Marquard S. L., Shan B., Brennaman M. K., Meyer G. J., Dares C., Meyer T. J. (2017). Water Photo-Oxidation Initiated by Surface-Bound Organic Chromophores. J. Am. Chem. Soc..

[cit21] Kafizas A., Xing X., Selim S., Mesa C. A., Ma Y., Burgess C., McLachlan M. A., Durrant J. R. (2019). Ultra-Thin Al_2_O_3_ Coatings on BiVO_4_ Photoanodes: Impact on Performance and Charge Carrier Dynamics. Catal. Today.

[cit22] Jurng S., Heiskanen S. K., Chandrasiri K. W. D. K., Abeywardana M. Y., Lucht B. L. (2019). Minimized Metal Dissolution from High-Energy Nickel Cobalt Manganese Oxide Cathodes with Al _2_ O _3_ Coating and Its Effects on Electrolyte Decomposition on Graphite Anodes. J. Electrochem. Soc..

[cit23] Eberhart M. S., Wang D., Sampaio R. N., Marquard S. L., Shan B., Brennaman M. K., Meyer G. J., Dares C., Meyer T. J. (2017). Water Photo-Oxidation Initiated by Surface-Bound Organic Chromophores. J. Am. Chem. Soc..

[cit24] Harari Y., Pathak C. S., Edri E. (2023). Molecular relays in nanometer-scale alumina: effective encapsulation for water-submersed halide perovskite photocathodes. Nanoscale.

[cit25] Harsha S., Sharma R. K., Dierner M., Baeumer C., Makhotkin I., Mul G., Ghigna P., Spiecker E., Will J., Altomare M. (2024). Dewetting of Pt Nanoparticles Boosts Electrocatalytic Hydrogen Evolution Due to Electronic Metal-Support Interaction. Adv. Funct. Mater..

[cit26] Boukamp B. A. (1995). A Linear Kronig–Kramers Transform Test for Immittance Data Validation. J. Electrochem. Soc..

[cit27] Boukamp B. A. (2004). Electrochemical impedance spectroscopy in solid state ionics: recent advances. Solid State Ionics.

[cit28] Biegler T., Rand D. A. J., Woods R. (1971). Limiting Oxygen Coverage on Platinized Platinum; Relevance to Determination of Real Platinum Area Hydrogen Adsorption. J. Electroanal. Chem..

[cit29] Katsoukis G., Heida H., Gutgesell M., Mul G. (2024). Time-Resolved Infrared Spectroscopic Evidence for Interfacial pH-Dependent Kinetics of Formate Evolution on Cu Electrodes. ACS Catal..

[cit30] Morterra C., Magnacca G. (1996). A Case Study: Surface Chemistry and Surface Structure of Catalytic Aluminas, as Studied by Vibrational Spectroscopy of Adsorbed Species. Catal. Today.

[cit31] Orazem M. E. and Tribollet B., Electrochemical Impedance Spectroscopy, Wiley, New Jersey, 2nd edn, 2017

[cit32] Franzetti I., Pushkarev A., Chan A.-L., Smolinka T. (2023). Parasitic Effects in Impedance Spectrum of PEM Water Electrolysis Cells: Case Study of High–Frequency Inductive Effects. Energy Technol..

[cit33] Hirschorn B., Orazem M. E., Tribollet B., Vivier V., Frateur I., Musiani M. (2010). Determination of Effective Capacitance and Film Thickness from Constant-Phase-Element Parameters. Electrochim. Acta.

[cit34] Chen Y., Wippermann K., Rodenbücher C., Suo Y., Korte C. (2024). Impedance Analysis of Capacitive and Faradaic Processes in the Pt/[Dema][TfO] Interface. ACS Appl. Mater. Interfaces.

[cit35] Caldararu M., Postole G., Carata M., Hornoiu C., Ionescu N. I., Ioujakova T., Redey A. (2003). Adsorption on Transition Aluminas from in Situ Capacitance Measurements. Appl. Surf. Sci..

[cit36] Horvai G. (1991). Relationship between Charge Transfer Resistance and Exchange Current Density of Ion Transfer at the Interface of Two Immiscible Electrolyte Solutions. Electroanalysis.

[cit37] Wang X., Ahluwalia R. K., Steinbach A. J. (2013). Kinetics of Hydrogen Oxidation and Hydrogen Evolution Reactions on Nanostructured Thin-Film Platinum Alloy Catalyst. J. Electrochem. Soc..

[cit38] Durst J., Siebel A., Simon C., Hasche F., Herranz J., Gasteiger H. A. (2014). New insights into the electrochemical hydrogen oxidation and evolution reaction mechanism. Energy Environ. Sci..

[cit39] Hirschorn B., Orazem M. E., Tribollet B., Vivier V., Frateur I., Musiani M. (2010). Determination of Effective Capacitance and Film Thickness from Constant-Phase-Element Parameters. Electrochim. Acta.

[cit40] Bunker B. C., Nelson G. C., Zavadil K. R., Barbour J. C., Wall F. D., Sullivan J. P., Windisch C. F., Engelhardt M. H., Baer D. R. (2002). Hydration of Passive Oxide Films on Aluminum. J. Phys. Chem. B.

[cit41] Choi Y., Kim H. J., Kim E., Kang H., Park J., Do Y. R., Kwak K., Cho M. (2023). ACS Appl. Mater. Interfaces.

[cit42] Bernt M., Gasteiger H. A. (2016). Influence of Ionomer Content in IrO_2_/TiO_2_ Electrodes on PEM Water Electrolyzer Performance. J. Electrochem. Soc..

[cit43] Ramos N. C., Medlin J. W., Holewinski A. (2023). Electrochemical Stability of Thiolate Self-Assembled Monolayers on Au, Pt, and Cu. ACS Appl. Mater. Interfaces.

[cit44] Mujtaba A., Janua N. K. (2015). Fabrication and Electrocatalytic Application of CuO@Al_2_O_3_ Hybrids. J. Electrochem. Soc..

[cit45] Li Z., Li R., Jing H. (2023). *et al.*, Blocking the reverse reactions of overall water splitting on a Rh/GaN–ZnO photocatalyst modified with Al_2_O_3_. Nat. Catal..

