# Supplementary material for: Molecularly modified ultrathin Al2O3 layers as proton-conductive, oxygen-impermeable nanomembranes for catalytic surfaces
Source: Nanoscale. 2026 Feb 5;18(13):6894–905. doi: 10.1039/d5nr04262c (PMC12907788; doi:10.1039/d5nr04262c)
Supplement: NR-018-D5NR04262C-s001 [file NR-018-D5NR04262C-s001.pdf]

Supplementary Information (SI) for Nanoscale.
This journal is © The Royal Society of Chemistry 2026

SUPPORTING INFORMATION

Molecularly modified ultrathin Al_2O_3 layers: proton-conductive, oxygen-impermeable nanomembranes for catalytic surfaces

Dalia C. Leon-Chaparro,[1] Christos Englezos, [1] Bastian Mei,[1,2] Guido Mul,[1] and Georgios Katsoukis[*1]*

[1]Department of Chemical Engineering, MESA+ Institute for Nanotechnology, Faculty of Science and Technology, University of Twente, Drienerlolaan 5, 7522 NB Enschede, The Netherlands

[2]Laboratory of Industrial Chemistry, Ruhr-University Bochum, Universitätsstraße 150, 44801 Bochum, Germany

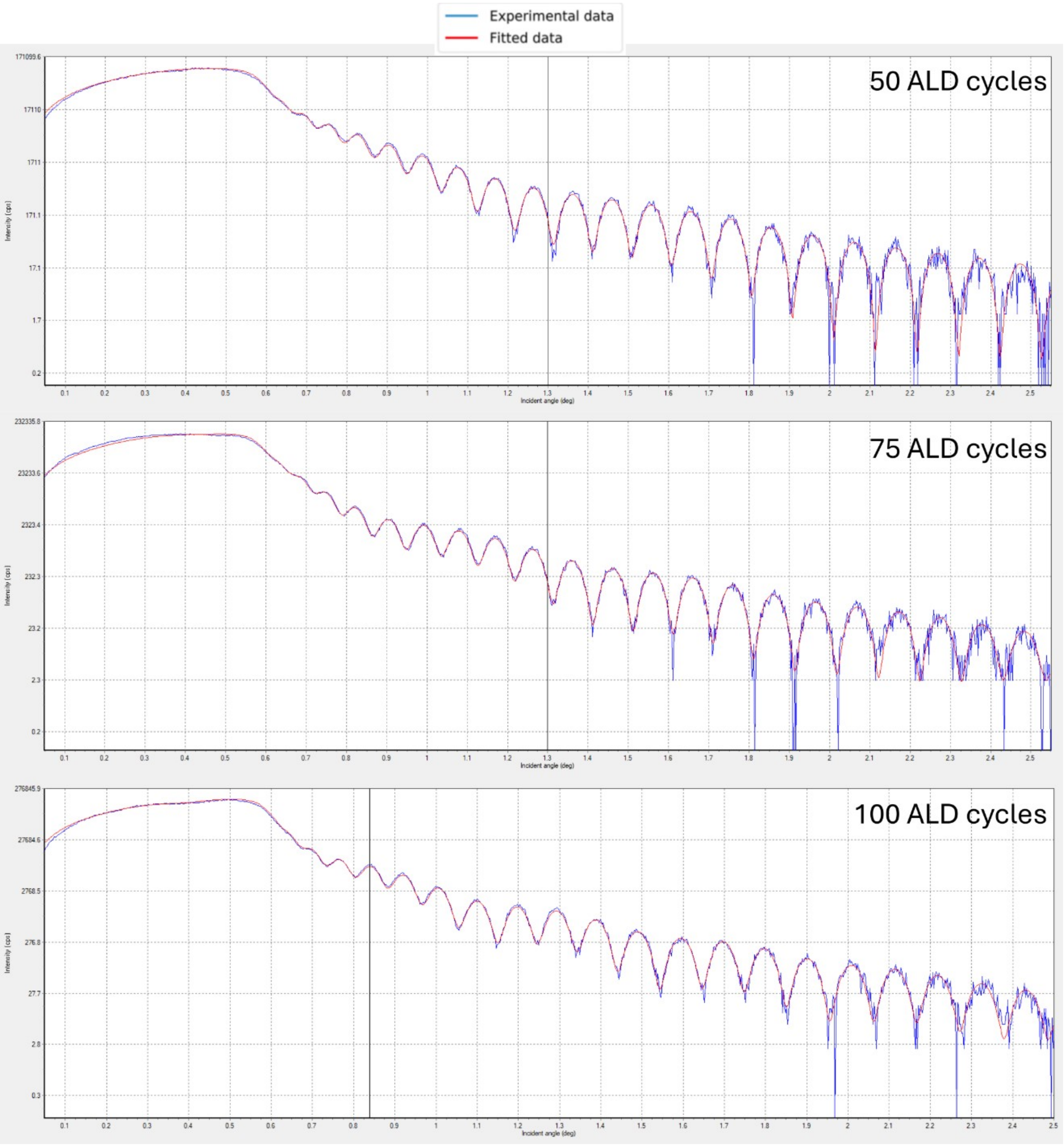

Figure S1: X-ray reflectivity (XRR) experimental data and corresponding fitted models for alumina films deposited by atomic layer deposition using trimethylaluminum and water for 50, 75, and 100 cycles. The fits were used to extract film thickness as shown in Figure S3. Measurements were performed using a Cu Kα radiation source (λ = 1.5406 Å). Experimental data were fitted using a multilayer slab model

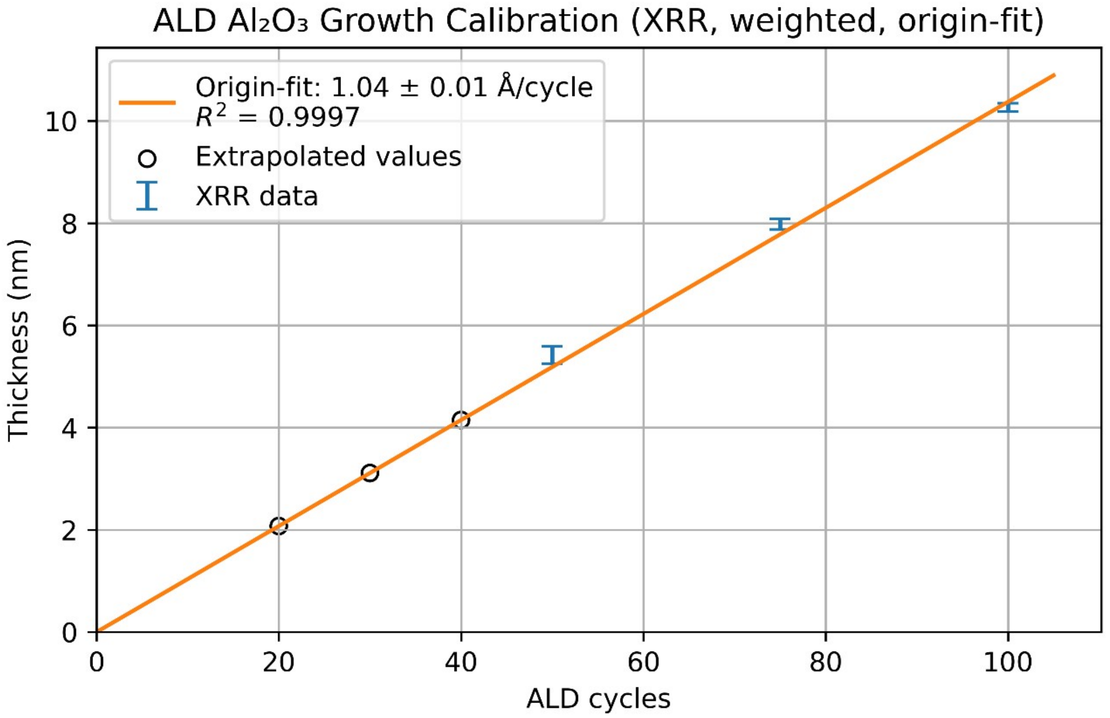

Figure S2: Growth calibration curve for atomic layer deposition of alumina using the XRR data in Figure S2.

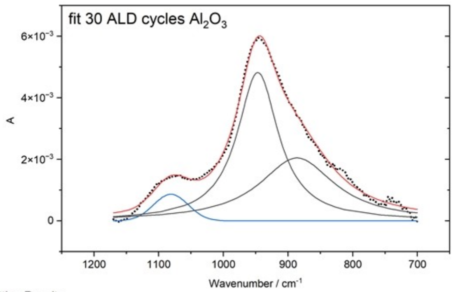

Fitting Results

Peak Index	Peak Type	Area Intg	FWHM	Max Height	Center Grvty	Area IntgP
1	Gaussian	0.05795	62.94583	8.65292E-4	1081.13273	6.11031
2	Lorentz	0.52439	77.31798	0.00482	946.79545	55.2905
3	Lorentz	0.36608	141.26301	0.00204	886.16361	38.59919

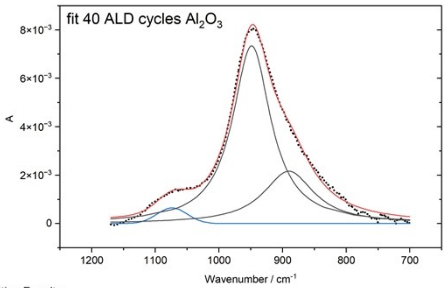

Fitting Results

Peak Index	Peak Type	Area Intg	FWHM	Max Height	Center Grvty	Area IntgP
1	Gaussian	0.03878	56.32807	6.46872E-4	1073.64385	3.58882
2	Lorentz	0.75696	72.80742	0.00734	948.72663	70.04243
3	Lorentz	0.28497	96.76789	0.00216	890.16628	26.36874

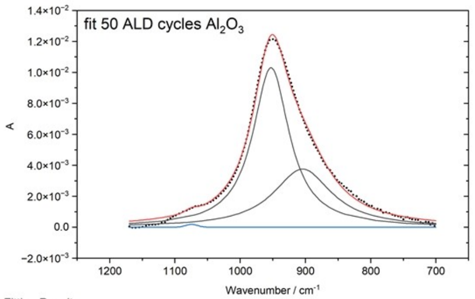

Fitting Results

Peak Index	Peak Type	Area Intg	FWHM	Max Height	Center Grvty	Area IntgP
1	Gaussian	0.00446	22.38868	1.87166E-4	1074.56152	0.28788
2	Lorentz	0.99575	67.69739	0.01031	952.84589	64.26401
3	Lorentz	0.54926	109.01155	0.00376	904.47153	35.44811

Figure S3: FT-IRRA spectra (dotted black line) with cumulative fit (red line), showing alumina-related contributions (grey Lorentzian components) and the aluminum hydroxide band (blue Gaussian component) for 30 (top left), 40 (top right), and 50 (bottom left) ALD cycles.

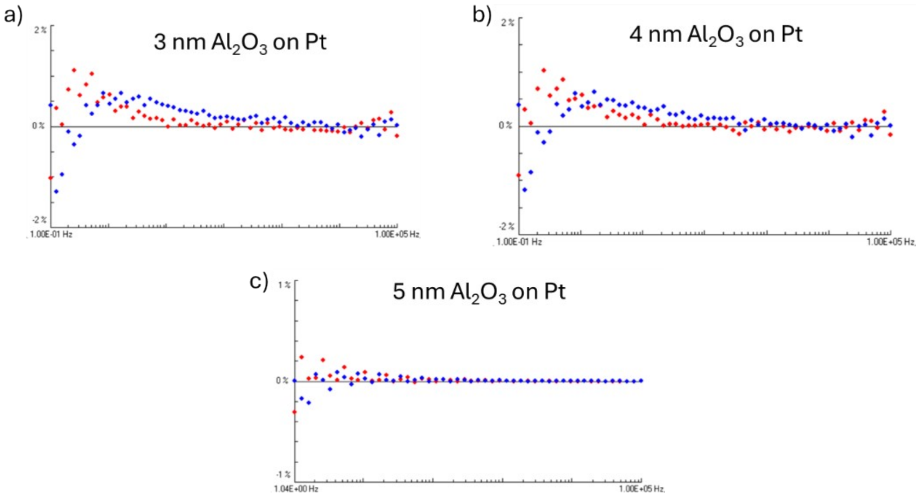

Figure S4: Kramers-Kronig test of the EIS data in Figure 2a.

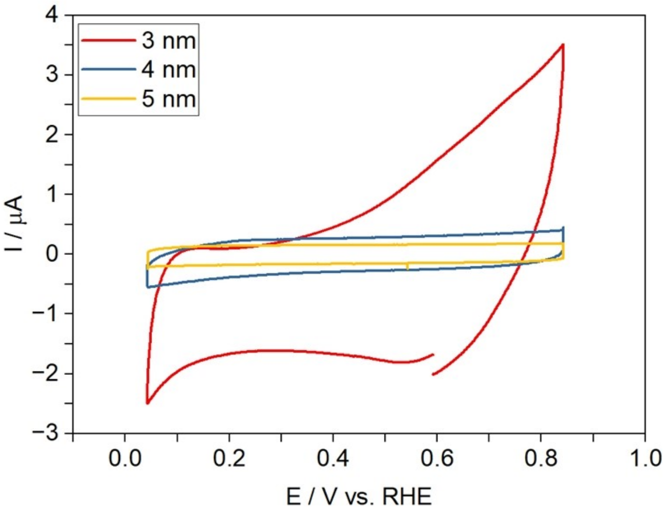

Figure S5: Cyclic voltammograms at 100 mV s^{-1} of Pt electrodes coated with 3, 4, and 5 nm alumina layers. The 3 nm sample was tested in ferro/ferricyanide solution to demonstrate its blocking behavior toward larger redox ions. These results are consistent with the EIS analysis, showing only a modest reduction in the electrical double-layer capacitance with increasing alumina thickness.

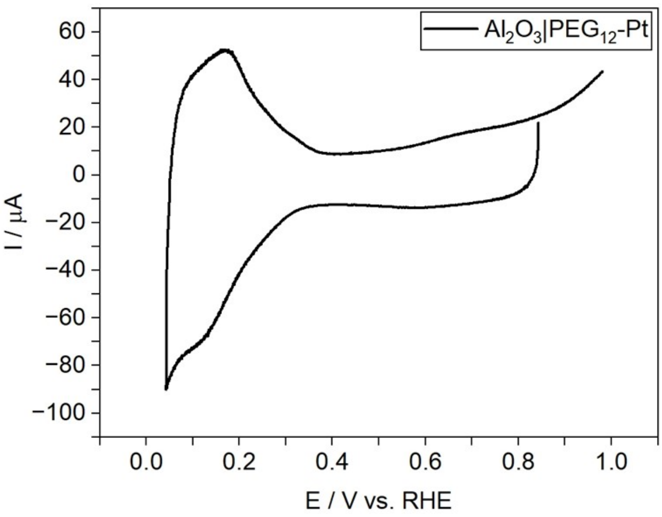

Figure S6: CV of 3 nm Al_2O_3|PEG_{10}-Pt after 10 cycles in 0.5 M Na_2SO_4 (pH 4), showing the significantly increased H_{upd} currents. The potential window was limited to below 0.9 V vs RHE to avoid anodic oxidation of the underlying Pt electrode, which could otherwise alter or rupture the alumina layer.

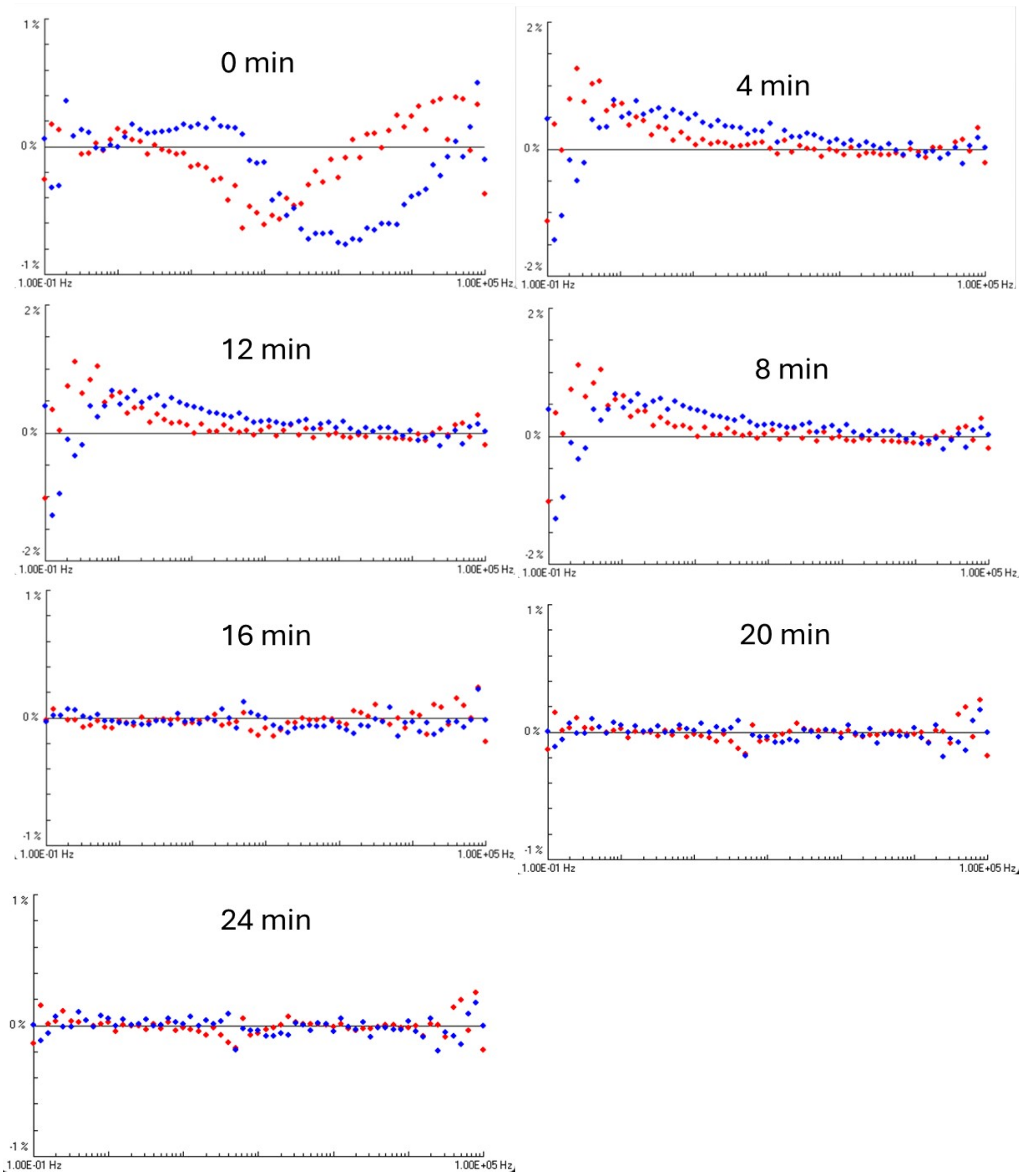

Figure S7: Kramers-Kronig tests for 3 nm Al2O3|PEG_{12}-Pt. The first KK test shows that the EIS data is far out of equilibrium.

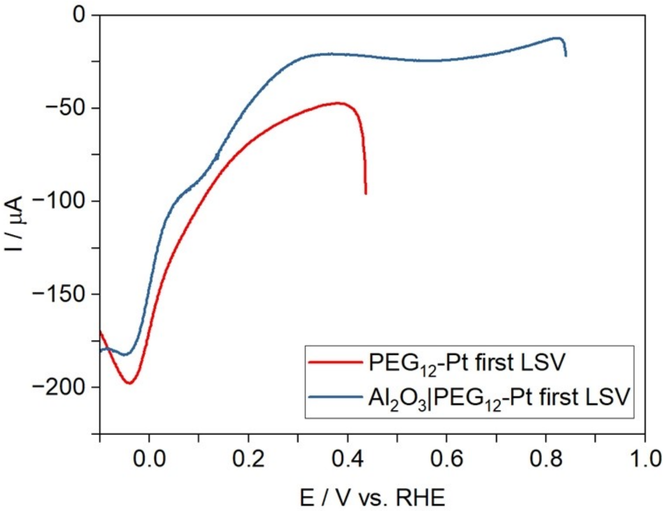

Figure S8: First linear sweep voltammogram starting from open circuit potential of 3 nm Al_2O_3|PEG_{10}-Pt and PEG_{10}-Pt in 0.5 M Na_2SO_4 (pH 4), highlighting the H_{upd} currents, into which the thiol stripping current is convoluted.

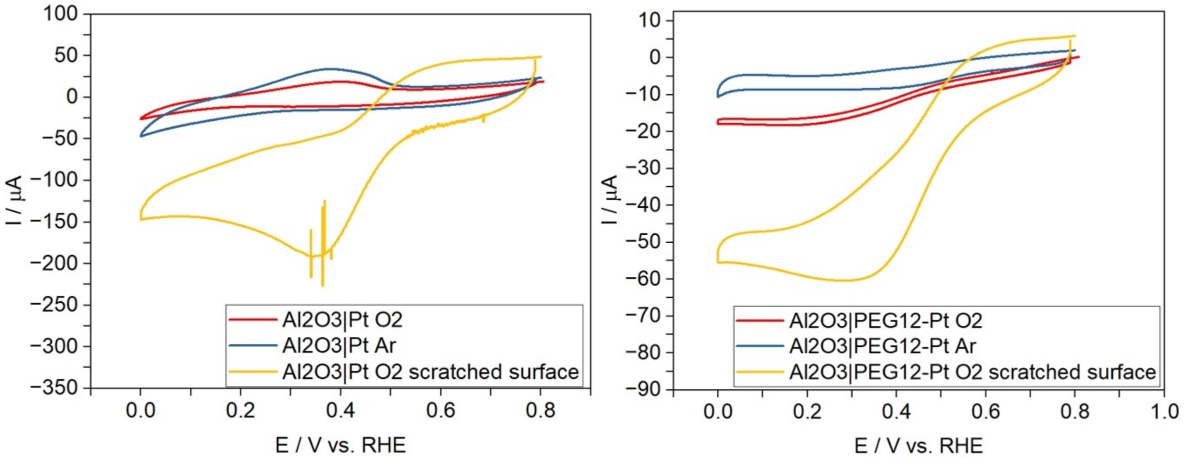

Figure S9: 10th CV of 3 nm Al_2O_3|Pt (left) and Al_2O_3|PEG_{12}-Pt (right) in O_2, Ar, and after scratching the surface in O_2.
